# Myocardial Connective Tissue Growth Factor (CCN2/CTGF) Attenuates Left Ventricular Remodeling after Myocardial Infarction

**DOI:** 10.1371/journal.pone.0052120

**Published:** 2012-12-20

**Authors:** Jørgen Gravning, Stein Ørn, Ole Jørgen Kaasbøll, Vladimir N. Martinov, Cord Manhenke, Kenneth Dickstein, Thor Edvardsen, Håvard Attramadal, Mohammed Shakil Ahmed

**Affiliations:** 1 Institute for Surgical Research, Oslo University Hospital, Rikshospitalet, Oslo, Norway; 2 Center for Heart Failure Research, University of Oslo, Oslo, Norway; 3 Division of Cardiology, Stavanger University Hospital, Stavanger, Norway; 4 Institute of Internal Medicine, University of Bergen, Bergen, Norway; 5 Department of Cardiology, Oslo University Hospital, Rikshospitalet, Oslo, Norway; Virginia Commonwealth University Medical Center, United States of America

## Abstract

**Aims:**

Myocardial CCN2/CTGF is induced in heart failure of various etiologies. However, its role in the pathophysiology of left ventricular (LV) remodeling after myocardial infarction (MI) remains unresolved. The current study explores the role of CTGF in infarct healing and LV remodeling in an animal model and in patients admitted for acute ST-elevation MI.

**Methods and Results:**

Transgenic mice with cardiac-restricted overexpression of CTGF (Tg-CTGF) and non-transgenic littermate controls (NLC) were subjected to permanent ligation of the left anterior descending coronary artery. Despite similar infarct size (area of infarction relative to area at risk) 24 hours after ligation of the coronary artery in Tg-CTGF and NLC mice, Tg-CTGF mice disclosed smaller area of scar tissue, smaller increase of cardiac hypertrophy, and less LV dilatation and deterioration of LV function 4 weeks after MI. Tg-CTGF mice also revealed substantially reduced mortality after MI. Remote/peri-infarct tissue of Tg-CTGF mice contained reduced numbers of leucocytes, macrophages, and cells undergoing apoptosis as compared with NLC mice. In a cohort of patients with acute ST-elevation MI (n = 42) admitted to hospital for percutaneous coronary intervention (PCI) serum-CTGF levels (s-CTGF) were monitored and related to infarct size and LV function assessed by cardiac MRI. Increase in s-CTGF levels after MI was associated with reduced infarct size and improved LV ejection fraction one year after MI, as well as attenuated levels of CRP and GDF-15.

**Conclusion:**

Increased myocardial CTGF activities after MI are associated with attenuation of LV remodeling and improved LV function mediated by attenuation of inflammatory responses and inhibition of apoptosis.

## Introduction

Myocardial infarction (MI) commonly triggers left ventricular (LV) remodeling, progressive deterioration of cardiac function, and ultimately, the clinical syndrome of heart failure (HF) [1]. However, current understanding of the pathophysiologic mechanisms of LV remodeling is still fragmentary. Thus, unraveling of autocrine/paracrine factors and cytokines involved in infarct healing and LV remodeling after MI may provide novel targets for pharmacologic therapeutics.

Myocardial connective tissue growth factor (CCN2/CTGF), a secreted matricellular protein of the CCN family, is upregulated in HF of both ischemic and non-ischemic etiologies, in experimental models as well as in humans [2], [3]. Plasma CTGF levels have also previously been reported to be elevated in HF patients and correlated to NYHA-class [4]. Although increased tissue expression of CTGF in disease is often associated with fibrosis [5], it is not yet known to what extent CTGF causes fibrosis. Thus, despite the unresolved role of elevated myocardial CTGF activities in heart failure, myocardial CTGF expression may reflect both fibrosis and functional derangement of the heart. A recent study reported preserved cardiac function in transgenic mice with cardiac-restricted overexpression of CTGF following chronic infusion of angiotensin II [6]. In a recent report from our laboratory, enhanced resistance towards ischemia/reperfusion injury was observed both in transgenic mice with cardiac-restricted overexpression of CTGF (Tg-CTGF) and in Langendorff-perfused hearts pre-treated with recombinant human CTGF (rh-CTGF) before ischemia, due to increased activity of the Akt/GSK-3β signaling pathway [7]. However, to what extent the cardioprotective effects of CTGF are maintained in chronic ischemia, infarct healing and LV remodeling remain to be investigated. Cardiac-restricted expression of CTGF resulted in minimal increase of myocardial fibrosis *per se*, and indicated that, at the least, additional co-factors are required for CTGF to stimulate relevant fibrosis [7]. Yet, to what extent MI provides basis for enhanced profibrotic actions of CTGF is unknown. No reports have yet explored the relationship between CTGF and LV remodeling following MI.

In this study we therefore investigated the role of CTGF in post-MI remodeling of the left ventricle both in an animal model of myocardial infarction and in a cohort of patients admitted for first-time, acute ST-elevation MI (STEMI) treated by primary PCI, and assessed with cardiac MRI and analysis of s-CTGF levels at several time points after the acute event. The current study unravels novel salutary actions of CTGF in infarct healing and LV remodeling after MI.

## Materials and Methods

All animal studies were performed in accordance with the NIH Guidelines for the Use of Laboratory Animals and were approved by the institutional and national boards for laboratory animal research. The patient study was approved by the Regional Ethics Committee of Western Norway and all patients provided written informed consent prior to inclusion.

### Experimental MI and Heart Failure in Mice

The transgenic mice with cardiac-restricted overexpression of CTGF (Tg-CTGF mice) employed in this study have been described and characterized previously [7]. Briefly, the Tg-CTGF mice were generated by pronuclear injection of a DNA cassette containing the ORF of rat CTGF cDNA under control of the mouse α-myosin heavy chain promoter into fertilized oocytes of C57BL/6 mice and implanted in pseudopregnant mice. Nontransgenic littermate control (NLC) mice were generated from non-injected C57BL/6 mice (siblings of the mice employed for pronuclear injection) and bred similarly to the transgenic lines, in order to ensure background similar to that of Tg-CTGF mice. Two transgenic lines that were propagated and characterized (Tg-CTGF/6 and Tg-CTGF/13) displayed similar phenotypic characteristics [7]. Unless otherwise indicated, Tg-CTGF/6 mice were employed in the experiments of the current study. MI was induced by ligation of the left anterior descending coronary artery (LAD) in Tg-CTGF (n = 22) and NLC mice (n = 21) according to previously published procedures [8]. Briefly, the mice were intubated, anesthetized with isoflurane and ventilated at a respiratory rate of 130/min in a gas mixture of 1/3 O_2_ and 2/3 N_2_O containing 1.5% isoflurane, using a rodent ventilator. The mice were immobilized on a heating pad and subjected to left-sided thoracotomy between the third and fourth intercostal space. After opening of the pericardium, ligation of LAD was made 1 mm under the left atrial appendage using a silk 7/0 suture. Pallor of the affected myocardium ensured ischemia. During follow-up, 8 Tg-CTGF mice and 13 NLC mice died. Separate groups of sham-operated Tg-CTGF mice (n = 5) and NLC mice (n = 6) underwent similar surgical procedure except ligation of LAD. The study end-point was 4 weeks (28 days) after induction of MI. Cardiac dimensions and cardiac function were assessed by a high-resolution ultrasonography system (Vevo 770, VisualSonics Inc., Toronto, Canada) using 2D-guided M-mode recording of the LV at the plane of the tip of the papillary muscle according to previously described methodology [7]. Transthoracic echocardiography of all Tg-CTGF mice (n = 22) and NLC mice (n = 21) to be subjected to MI was performed immediately before induction of MI (baseline; day -1). Thereafter, successive transthoracic echocardiography of all mice alive was performed 2 weeks after induction of MI (Tg-CTGF [n = 15]; NLC [n = 9]) and at study end-point (Tg-CTGF [n = 14]; NLC [n = 8]). At study end-point, LV function was also assessed by trans-carotid catheterization of the left ventricle and continuous recording of LV pressure, as previously described [7]. Separate groups of Tg-CTGF mice (n = 8) and NLC mice (n = 8) were subjected to ligation of LAD and subsequent perfusion with 1% Evans blue dye in order to assess the area at risk (AAR) in the two groups. LV sections were subjected to computerized planimetry in order to determine the area of non-perfused myocardial tissue. Another group of Tg-CTGF mice (n = 6) and NLC mice (n = 6) were subjected to permanent ligation of LAD in order to estimate area of myocardial necrosis (infarct size) relative to area at risk after 24 hours of ischemia. After 24 hours the mice were euthanized by cardioplegic arrest following infusion of KCl. The hearts were excised and perfused with 1% Evans blue dye, and 1 mm thick sections of the left ventricle were immediately prepared and stained with 2,3,5-triphenyltetrazolium chloride (TTC) for 20 min at 37°C. Finally, the LV sections were fixed in 4% paraformaldehyde and washed with PBS before computerized planimetry for determination of area of necrosis (infarction; area not stained with TTC) relative to area at risk (AAR; area not stained with Evans blue dye) as described previously [7].

Histochemistry and morphometric analysis of myocardial sections, determination of myocardial mRNA levels by real-time qPCR, Western blot analysis, and assay of myocardial hydroxyproline contents were performed as previously described [7]. Myocardial cells undergoing apoptosis were assessed by in situ terminal deoxynucleotidyl transferase-mediated digoxigenin-conjugated dUTP nick end labeling (TUNEL) of apoptotic nuclei (*In Situ* Cell Death Detection kit; Roche Diagnostics, Inc.) and nuclear co-staining with Hoechst 33258. The cross-sectional area of cardiac myocytes was determined in transverse sections of the left ventricle after staining of plasma membrane with rhodamine-labeled wheat germ agglutinin (WGA) (Vector Laboratories, Inc.). The cross-sectional area of individual myocytes was determined by digital planimetry using the Adobe Photoshop software.

### Immunohistochemical Analysis of Myocardial Tissue Sections

Hearts from NLC CHF (*n* = 4) and Tg-CTGF CHF (*n* = 4) mice were fixed in 4% paraformaldehyde in phosphate-buffered saline for 1 h, embedded in paraffin wax, and stored at 4°C. Immunohistochemical analysis of myocardial sections (6 *µ*m) of NLC and Tg-CTGF mice was performed as previously described [7], using rat monoclonal anti-mouse CD68 (ab53444; Abcam, Inc.), rabbit polyclonal anti-mouse CD45 IgG (ab10558; Abcam, Inc.), rabbit anti-c-Kit IgG (sc-5535; St. Cruz Biotechnology, Inc.), rabbit polyclonal anti-mouse Ki-67 IgG (ab9260; Millipore, Inc.) and goat polyclonal anti-mouse PECAM-1/CD31 IgG (sc-1506; St. Cruz Biotechnology, Inc.). The avidin-biotin-peroxidase system (Vectastain Elite kit; Vector Laboratories, Inc.) was used for signal amplification.

### The Patient Study

Forty-two consecutive patients admitted with ST-elevation MI (STEMI) and selected for primary PCI were enrolled prospectively at a single center [9]. Patients were included if they had: (i) no previous MI, (ii) demonstrated acute proximal/mid-occluded single-vessel disease, (iii) underwent successful PCI with stent implantation without significant residual stenosis, (iv) had no contraindications to CMR imaging, and (v) could be scanned within 48 h following PCI.

### Blood Sample Protocol and Assay of Serum CTGF, CRP and GDF-15 Levels

Venous blood samples were collected in pyrogen-free ethylenediaminetetraacetic acid (EDTA)-containing tubes or tubes without any additives at the time of the CMR examinations, at day 2, 1 week, 2 months and 1 year following PCI. The tubes were subsequently centrifuged at 1000x*g* for 10 min for preparation of plasma and serum samples, respectively. All samples were stored at –80°C until analysis. Serum levels of CTGF (s-CTGF) were determined by a sandwich enzyme-linked immunosorbent assay (ELISA) as previously described [10]. Serum levels of high-sensitive C-reactive protein (CRP) and Growth differentiation factor 15 (GDF-15) were determined by ELISA assays according to previously described methodology [11], [12].

### Cardiac Magnetic Resonance Protocol

All patients were scanned 2 days (mean±SEM: 2.2±0.1 days) following PCI, and subsequently 7 days (7.3±0.1 days), 2 months (61±0.6 days), and 1 year (364±1.2 days) following PCI, using a 1.5 T whole body scanner (Intera R 10.3 Philips Medical Systems, Best, The Netherlands) as previously described [9].

### Statistics

Continuous data are presented as mean±SEM and inter-group comparisons were made by two-tailed unpaired Student’s *t* test or 2-way ANOVA with post-hoc analysis (Bonferroni correction) as appropriate. Categorical variables are expressed as proportions and comparisons were made by two-sided Chi-Square test. Survival analysis was evaluated with Kaplan-Meier curves and compared by log-rank test. Intra-group changes in the CMR parameters during follow-up were analyzed with the Friedman test. *P*-value <0.05 was considered statistically significant for inter-group comparisons and *P*-value <0.01 was considered statistically significant for intra-group variation.

## Results

### Tg-CTGF Mice and NLC Mice Displayed Similar Area at Risk after Ligation of LAD

**Figure 1:**
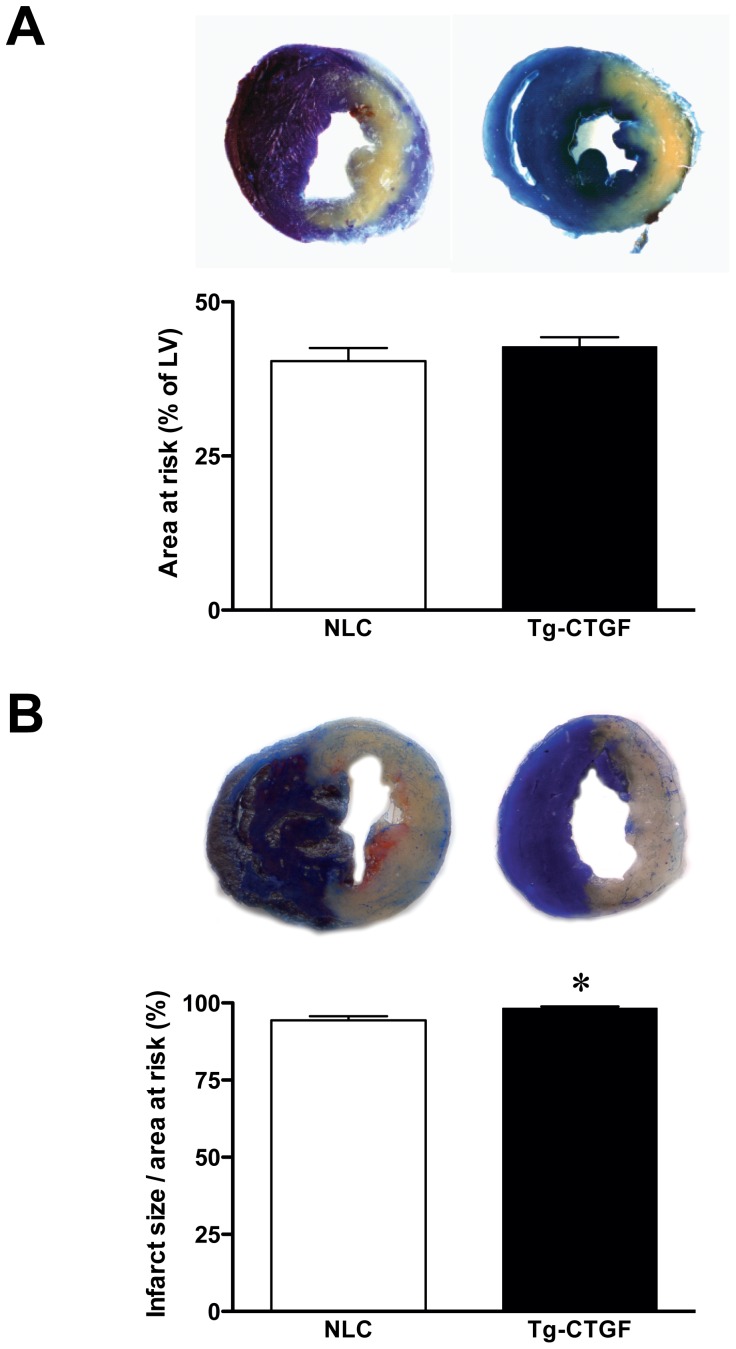
Area at risk and infarct size in NLC and Tg-CTGF mice subjected to ligation of LAD. A. Photomicrographs of representative myocardial sections of NLC and Tg-CTGF hearts perfused with Evans blue dye immediately after ligation of LAD. Histogram demonstrating area at risk in the left ventricles of NLC and Tg-CTGF mice determined by computerized planimetry after excision of the hearts. The data are mean±SEM of area at risk as percent of total LV area of NLC (n = 8) and Tg-CTGF mice (n = 8), respectively (40.4±2.1% vs. 42.7±1.6%, *P* = 0.39). B. Photomicrographs of representative myocardial sections of NLC and Tg-CTGF hearts 24 hours after ligation of LAD and subsequent perfusion with Evans blue dye and myocardial staining with TTC. Histogram demonstrating infarct size (area of necrosis relative to area at risk) 24 hours after ligation of LAD, determined as described in the Materials and Methods section. The data are mean±SEM of NLC (n = 6) and Tg-CTGF hearts (n = 6). *P<0.05 vs. NLC group.

As shown in Fig. 1A, hearts from Tg-CTGF and NLC mice perfused with Evans blue dye immediately after ligation of LAD displayed similar area at risk (42.7±1.6%, n = 8 vs. 40.4±2.1%, n = 8, *P* = 0.39).

### Similar Infarct Size in Tg-CTGF and NLC Mice 24 Hours after Ligation of LAD

Infarct size, i.e. area of myocardial necrosis relative to area at risk, 24 hours after ligation of LAD involved almost entire area at risk and was essentially similar in Tg-CTGF and NLC mice (Tg-CTGF: 98.3±0.5%, n = 6 vs. NLC: 94.4±1.3%, n = 6, *P* = 0.02) (Fig. 1B).

### CTGF Attenuates LV Dysfunction, Congestive Heart Failure, and Mortality after MI in Mice

**Figure 2:**
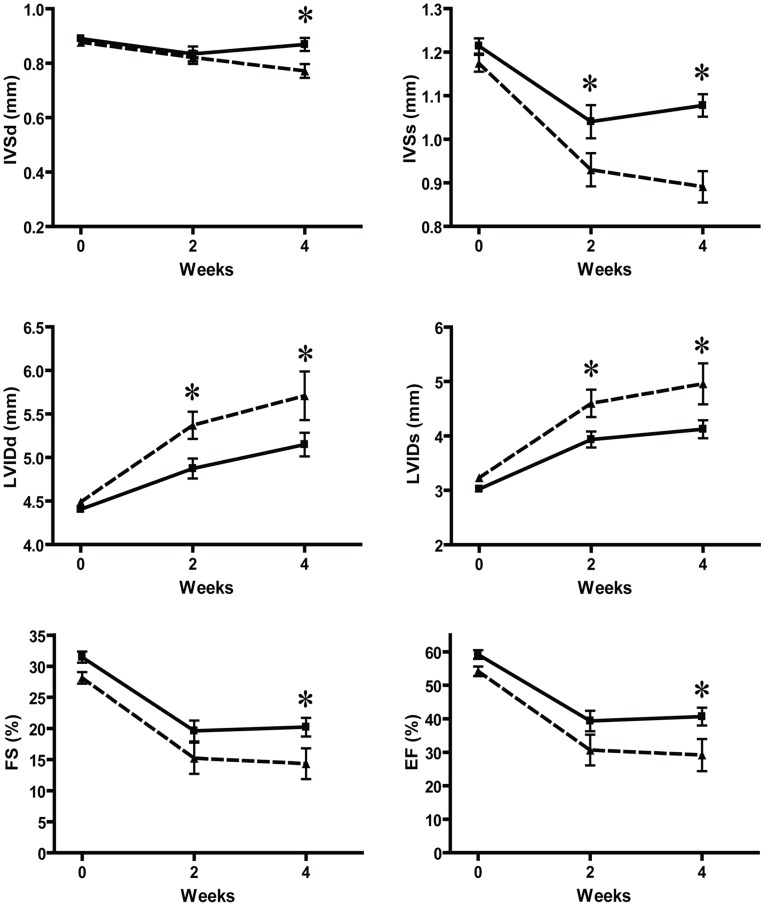
Transthoracic echocardiography of NLC and Tg-CTGF mice at successive time points after MI. Successive echocardiographic recordings of inter-ventricular septum thickness (IVS) and LV transverse diameter (LVID) at end-diastole and end-systole, as well as fractional shortening (FS) and estimated ejection fraction (EF) in the total number of NLC CHF (▴) and Tg-CTGF CHF mice (▪), at baseline and 2 and 4 weeks after ligation of LAD. Values are mean±SEM of NLC CHF and Tg-CTGF CHF mice. *P<0.05 vs. NLC group.

Cardiac dimensions at baseline assessed by transthoracic echocardiography were similar among the groups. Four weeks after MI, Tg-CTGF mice displayed diminished LV remodeling with less LV dilatation and less thinning of the intraventricular septum thickness both at end-diastole (IVSd) and at end-systole (IVSs) compared with NLC mice (Fig. 2). The decline of LV fractional shortening (FS) and LV ejection fraction (EF) were substantially reduced in Tg-CTGF mice versus NLC mice during the study period.

**Figure 3:**
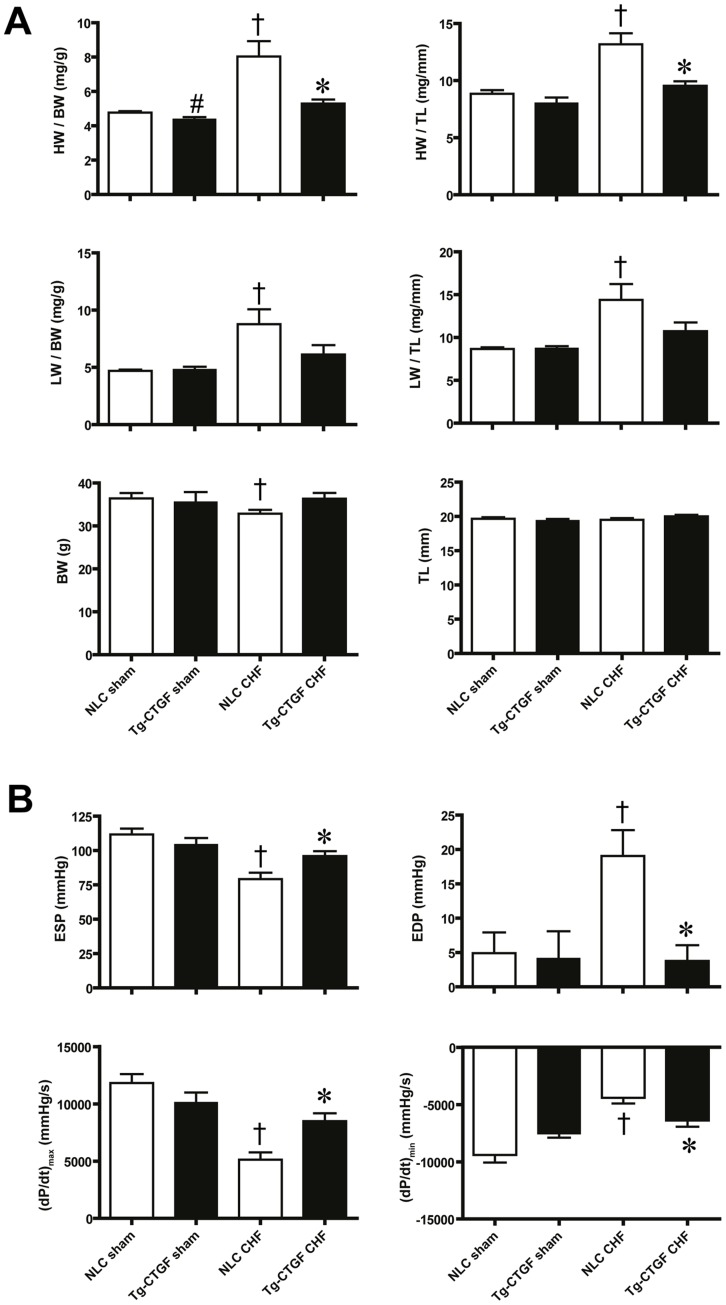
Gravimetric analyses and assessments of left ventricular pressures in NLC and Tg-CTGF mice after MI. A. Histograms of cardiac mass (HW) and dry lung weight (LW) normalized to body weight (BW) or tibia length (TL) in NLC CHF (n = 8) and Tg-CTGF CHF (n = 14) 4 weeks after ligation of LAD and in corresponding NLC sham (n = 6) and Tg-CTGF sham (n = 5) animals. B. LV end-systolic pressure (ESP), end-diastolic pressure (EDP) and the contractile parameters (dP/dt)_max_ and (dP/dt)_min_ in NLC CHF (n = 8) vs. Tg-CTGF CHF mice (n = 11) subjected to LV catheterization. Data are presented as mean±SEM. *P<0.05 vs. NLC CHF mice, ^#^ *P*<0.05 vs. NLC sham, ^†^ *P*<0.05 vs. corresponding sham group.

At study end-point gravimetric analysis revealed substantially smaller increase of cardiac mass in Tg-CTGF compared with NLC mice (Fig. 3A). In NLC mice subjected to MI, normalized lung weights were substantially increased at study end-point compatible with pulmonary congestion. In contrast, Tg-CTGF mice did not display significant alterations of lung weights from baseline to study end-point. Hemodynamic analysis of cardiac function by LV catheterization at study end-point, revealed substantial decline of end-systolic pressure (ESP) and increase of end-diastolic pressure (EDP) in NLC mice subjected to MI. In contrast, only subtle derangements of ESP and EDP were observed in Tg-CTGF mice at study end-point. Consistently, cardiac contractility as measured by LV (dP/dt)_max_ and (dP/dt)_min_ was less deteriorated in Tg-CTGF versus NLC mice (Fig. 3B).

**Figure 4:**
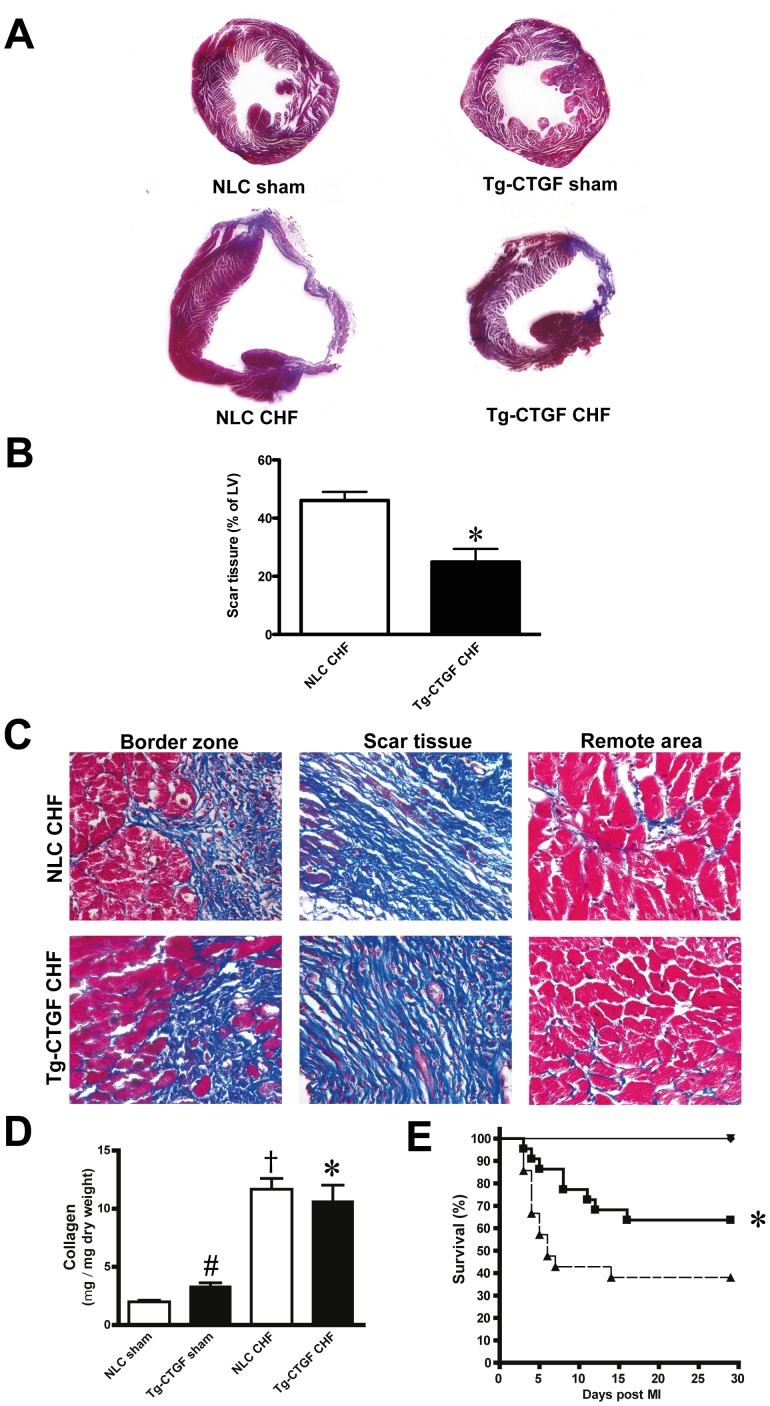
Assessments of survival and myocardial fibrosis after MI in NLC and Tg-CTGF mice. A. Transverse sections of the left ventricle (plane of papillary muscle) of hearts from NLC CHF and Tg-CTGF CHF mice 4 weeks after MI, and of hearts from corresponding sham animals, stained with Massońs trichrome stain. B. Representative images of Massońs trichrome-stained sections from border zone, scar tissue and remote area in Tg-CTGF CHF and NLC CHF mice. Magnification: ×400. C. Histograms demonstrating area of scar tissue as percentage of left ventricular area in NLC CHF (n = 4) and Tg-CTGF CHF mice (n = 4) 4 weeks after MI. Data are presented as mean±SEM. *P<0.05 vs. NLC CHF mice. D. Histogram demonstrating quantification of myocardial fibrosis by assay of myocardial collagen (hydroxyproline) by quantitative HPLC of HCl-hydrolyzed non-ischemic myocardial tissue 4 weeks after MI in NLC CHF (n = 6) vs. Tg-CTGF CHF mice (n = 11). Data are presented as mean±SEM. *P<0.05 vs. NLC CHF mice, ^#^ *P*<0.05 vs. NLC sham, ^†^ *P*<0.05 vs. corresponding sham group. E. Kaplan-Meier plot demonstrating survival rates of Tg-CTGF mice (▪) versus NLC mice (▴) after MI. There were no deaths among sham animals (▾).

Masson’s trichrome staining of transverse myocardial sections at the level of the papillary muscles revealed substantially lesser scar tissue in Tg-CTGF versus NLC mice at study end-point (Fig. 4A–B). Representative images of Masson’s trichrome-stained sections from the border zone, scar tissue and remote area of Tg-CTGF mice and NLC mice are displayed in Fig. 4C. Quantification of myocardial fibrosis in the remote area by assay of myocardial collagen (hydroxyproline), revealed similar collagen contents in Tg-CTGF and NLC mice after MI (Fig. 4D). However, the increase of myocardial collagen contents after MI relative to the respective sham group was smaller in Tg-CTGF than in NLC mice. Importantly, during the course of the study the survival rate was significantly higher among Tg-CTGF mice than NLC mice (64% vs. 38%, *P*<0.05) after MI (Fig. 4E).

### Assessment of Cardiomyocyte Hypertrophy, Myocardial Apoptosis, and Microvessel Densities in Tg-CTGF and NLC Mice after MI

**Figure 5:**
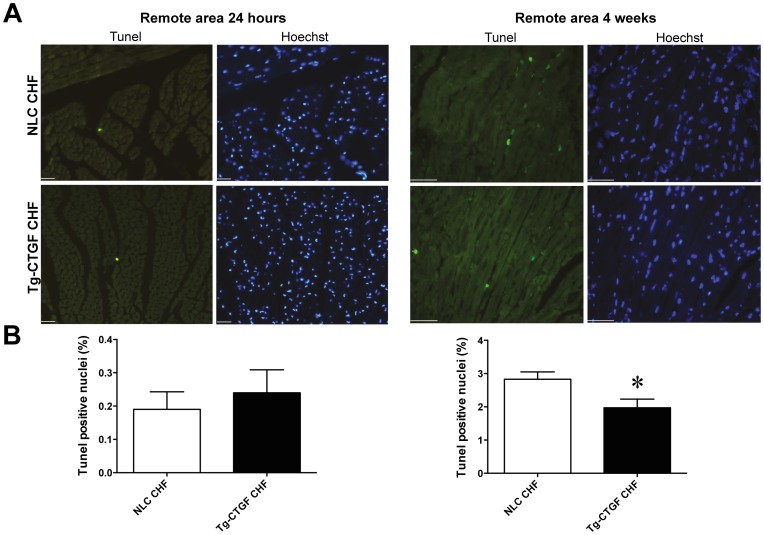
Morphometric analysis of myocardial cells undergoing apoptosis in NLC and Tg-CTGF mice after MI. A. Representative photomicrographs of myocardial sections subjected to staining of cells undergoing apoptosis in remote myocardium of NLC CHF and Tg-CTGF CHF mice 24 hours and 4 weeks after MI. Sections are stained with TUNEL assay and Hoechst as detailed in the Materials and Methods section. Size bar indicates 100 µm. B. Histograms of TUNEL positive nuclei in the remote myocardium of NLC CHF and Tg-CTGF CHF mice 24 hours and 4 weeks after ligation of LAD. Two visual fields/section and 3 sections per mice were analyzed. Data are mean±SEM of Tg-CTGF CHF (n = 4) and NLC CHF mice (n = 4). *P<0.05 vs. NLC CHF group.

Assessment of myocardial apoptosis by TUNEL-staining of apoptotic nuclei revealed very rare events of cellular apoptosis in the remote myocardium 24 hours after induction of MI. Cellular apoptosis in remote myocardium at this time point was not statistically different in Tg-CTGF and NLC mice (Fig. 5A–B). However, 4 weeks after MI cellular apoptosis of remote myocardial tissue was substantially increased. Yet, apoptosis of remote myocardial tissue was significantly lower in Tg-CTGF mice than in NLC mice at the later time point (Fig. 5A–B).

**Figure 6:**
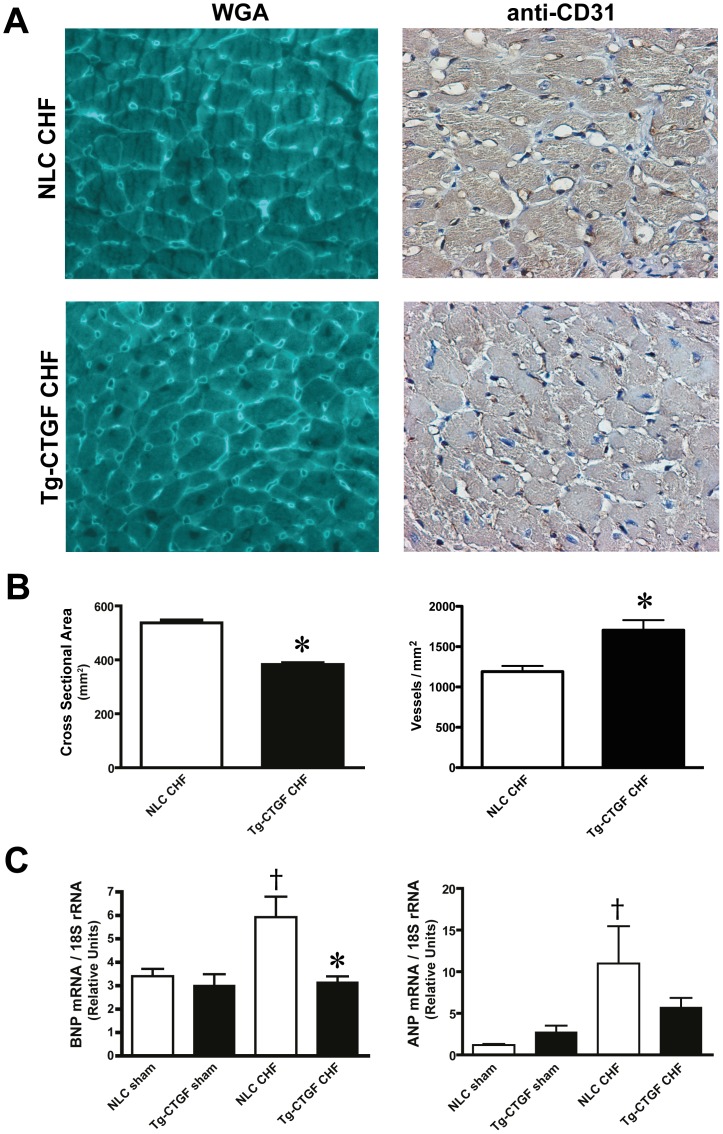
Cardiomyocyte cross-sectional area, microvessel densities, and mRNA expression markers of myocardial hypertrophy after MI. A. Photomicrographs of representative transverse sections of NLC CHF and Tg-CTGF CHF mice 4 weeks after MI stained with WGA or anti-mouse CD31 IgG. B. Histograms demonstrating cardiac myocyte cross-sectional areas and microvessel densities in NLC CHF vs. Tg-CTGF CHF mice 4 weeks after MI determined in transverse myocardial sections after staining with rhodamine-labeled WGA and immunohistochemical staining with anti-CD31, respectively. C. Myocardial BNP and ANP mRNA levels 4 weeks after MI in NLC CHF (n = 6) and Tg-CTGF CHF mice (n = 11) versus respective sham groups. All values are presented as mean±SEM. *P<0.05 vs. NLC CHF mice, ^†^ *P*<0.05 vs. corresponding sham group.

Consistent with the echocardiographic and hemodynamic data demonstrating attenuated remodeling and preserved cardiac function in Tg-CTGF versus NLC mice after MI, morphometric analysis of transverse myocardial sections stained with rhodamin-labeled WGA revealed decreased cross-sectional area of cardiac myocytes of Tg-CTGF mice versus NLC (Fig. 6A–B). Reflecting reduced signals for myocardial hypertrophy of Tg-CTGF mice after MI, the increase of myocardial BNP and ANP mRNA levels were also attenuated in Tg-CTGF mice, compared with that of NLC mice after MI (Fig. 6C). Importantly, mRNA expression of the rat-CTGF transgene in myocardial tissue of Tg-CTGF mice was not altered after MI excluding alterations of α-MHC promoter activities after MI (data not shown). Staining of myocardial tissue with anti-CD31 antibody displayed slight increase of microvessel density in the peri-infarct region of Tg-CTGF mice (Fig. 6A–B).

### Myocardial Tissue of Tg-CTGF Mice Display Decreased Contents of Inflammatory Cells after MI

**Figure 7:**
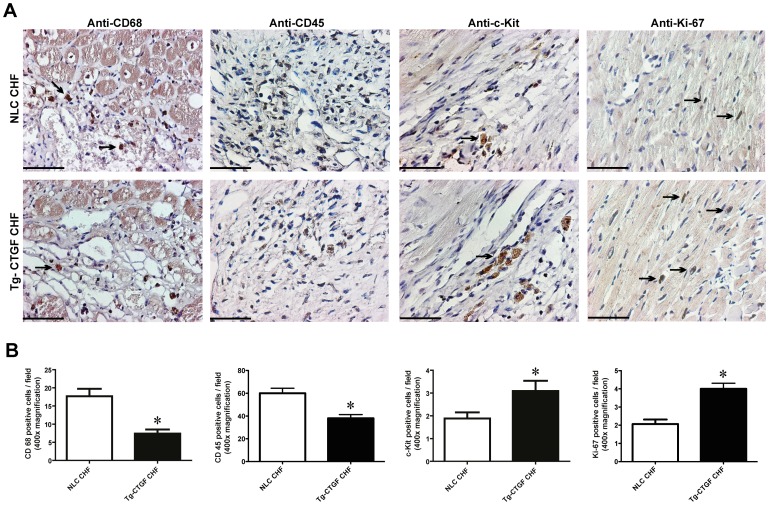
Morphometric analyses of inflammatory cells and cells undergoing apoptosis in myocardial tissue after MI. Photomicrographs of immunohistochemical staining of CD68, CD45, c-Kit and Ki-67 in myocardial sections of hearts from NLC mice and Tg-CTGF mice 4 weeks after MI. Panels are from border zone of MI. Size bar indicates 50 µm. Arrows indicate examples of immunoreactive cells. Magnification: ×400. B. Histograms of CD68^+^-cells, CD45^+^-cells, c-kit^+^-cells and Ki-67^+^-cells (immunoreactive cells/400x power field) in peri-infarct region of NLC CHF and Tg-CTGF CHF mice 4 weeks after ligation of LAD. 5 visual fields/section and 3 sections per mice were analyzed. Data are mean±SEM of Tg-CTGF CHF (n = 4) and NLC CHF mice (n = 4). *P<0.05 vs. NLC CHF group.

Immunostaining against the macrophage marker CD68 and the leucocyte marker CD45 also revealed decreased contents of macrophages and leucocytes in peri-infarct tissue of hearts from Tg-CTGF mice compared with hearts from NLC mice at study end-point 4 weeks after MI (Fig. 7).

### Myocardial Tissue of Tg-CTGF Mice Display Increased Numbers of c-kit^+^- and Ki-67^+^-Cells after MI

As shown in Fig. 7, the peri-infarct region of hearts from Tg-CTGF mice revealed increased numbers of c-kit^+^- and Ki-67^+^-cells compared with that of hearts from NLC mice 4 weeks after MI, indicating increased stem cell activity and increased number of proliferating cells in the myocardium of mice overexpressing CTGF.

### Serum CTGF Levels after MI in STEMI Patients

**Figure 8:**
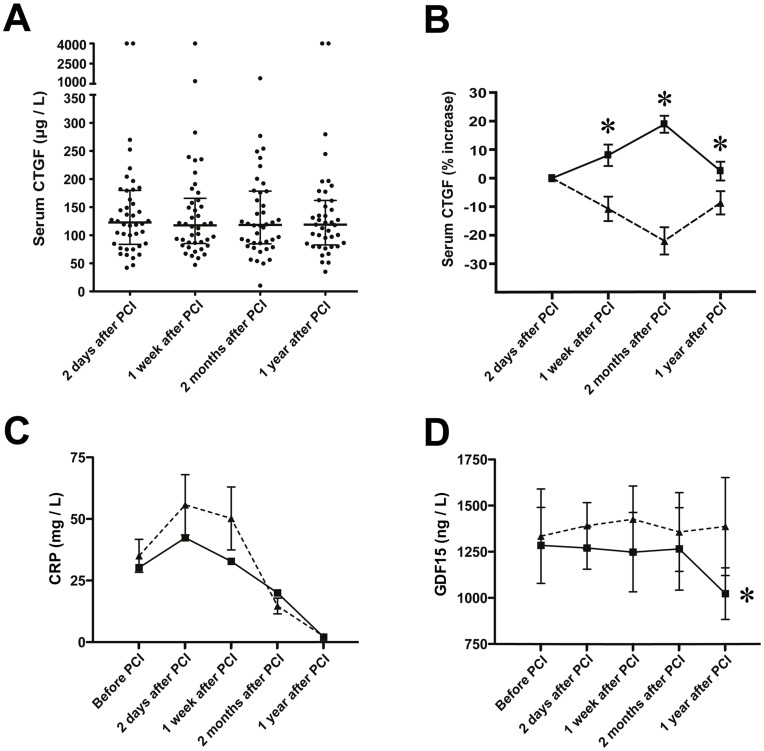
Serum levels of CTGF, CRP and GDF-15 in patients after myocardial infarction. A. Scatter-plot of serum CTGF levels at various time points after percutaneous coronary intervention in patients (n = 42) admitted for acute ST-elevation MI. Median serum CTGF levels and interquartile range (25^th^ to 75^th^ percentile) at each time point are indicated. B. Time course of s-CTGF levels after stratification of the patient cohort into two groups: patients that displayed increase in s-CTGF levels after MI (▪; n = 21), and patients in which s-CTGF levels remained unaltered or declined after MI (▴; n = 21). The figure demonstrates s-CTGF levels plotted as percent change from day 2 after PCI for each time point during the one-year follow-up after MI. Values are mean±SEM. *P<0.05 between groups. C–D. Panels demonstrating serial measurements of serum levels of CRP and GDF-15 in the two patient groups. Values are mean±SEM. *P<0.01 for intra-group comparisons.

S-CTGF levels were determined in 42 patients with acute ST-elevation MI, 2 days, one week, 2 months, and 1 year after PCI. No overall changes of s-CTGF levels were observed throughout the study period (Fig. 8A). However, significant intra-patient changes were observed during the time course of the study. Therefore, patients were stratified according to those in which s-CTGF levels increased after MI versus those in which s-CTGF levels decreased or remained unaltered. 21 patients displayed an increase of s-CTGF levels, whereas 21 patients displayed unchanged or lower s-CTGF levels after MI (Fig. 8B). These groups were used to assess the relationship between s-CTGF and cardiac remodeling as evaluated by CMR.

### Similar Baseline Characteristics among Patients with and without Increased s-CTGF Levels after MI

Baseline characteristics for patient groups are shown in Table 1. There were no statistical differences for any of the listed parameters, except for cholesterol levels.

**Table 1:** Baseline characteristics of patients with or without increase of serum-CTGF levels after MI.

	No s-CTGF increase after MI	s-CTGF increase after MI	
	n = 21	n = 21	p
			
Age at inclusion (years)	59±3	58±2	ns
Male sex	17 (81%)	17 (81%)	ns
Body mass index (kg/m^2^)	27±1	27±1	ns
Body surface area (m^2^)	2.01±0.04	2.00±0.05	ns
Systolic blood pressure (mmHg)	142±7	138±6	ns
Diastolic blood pressure (mmHg)	88±4	80±5	ns
Heart rate (min^−1^)	77±5	70±3	ns
**History**			
Pre-infarction angina	4 (19%)	9 (42%)	ns
Current smoker	8 (38%)	11 (52%)	ns
Diabetes mellitus	2 (9.5%)	1 (4.8%)	ns
Hypertension	5 (24%)	5 (24%)	ns
**Labs**			
Cholesterol (mmol/L)	5.3±0.2	6.0±0.3	0.04
Troponin-T (µg/L)	0.16±0.06	0.15±0.05	ns
Creatinine (µmol/L)	74±4	75±3	ns
GFR (mL/min)	100±7	98±5	ns
**Procedural data**			
LAD lesion	13 (62%)	8 (38%)	ns
CX lesion	3 (14%)	3 (14%)	ns
RCA lesion	5 (24%)	10 (48%)	ns
Lesion location (proximal/mid/distal)	8/12/1	10/9/2	ns
Gp IIb/IIIa antagonist	16 (76%)	15 (71%)	ns
Symptom-flow time (min)	263±41	250±40	ns
TIMI flow post-PCI (3/2/1)	18/3/0	17/4/0	ns
**Discharge medications**			
ASA	21 (100%)	21 (100%)	ns
Beta-blocker	9 (43%)	11 (52%)	ns
ACEI/ARB	19 (91%)	19 (91%)	ns
Statin	21 (100%)	21 (100%)	ns
Aldosterone antagonist	5 (24%)	2 (10%)	ns
Clopidogrel	21 (100%)	21 (100%)	ns

### Increase in s-CTGF Levels in Patients after MI was Associated with Attenuation of CRP and GDF-15

In the early phase after myocardial infarction, patients with increased levels of s-CTGF tended to have an attenuation of CRP levels, although the differences did not reach statistical significance (Fig. 8C). However, serum levels of GDF-15 were significantly reduced during the study period in these patients, which was not the case for patients with unchanged or decreased s-CTGF levels after PCI (Fig. 8D).

### Attenuation of Cardiac Remodeling in Patients with Increased s-CTGF Levels after MI

**Figure 9:**
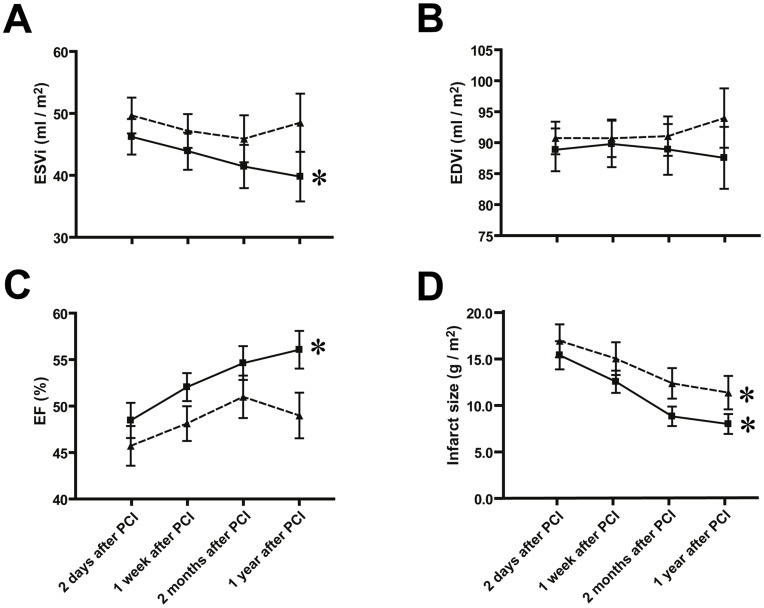
Serial assessments of left ventricular volumes and infarct size in patients stratified according to s-CTGF levels after MI. Panels demonstrating end-systolic volume index (ESVi) (A), end-diastolic volume index (EDVi) (B), ejection fraction (EF) (C) and infarct size (D) determined by CMR at successive time points from 2 days to 1 year after PCI in patients who responded with increase in s-CTGF levels after MI (▪; n = 21) compared with those in whom s-CTGF levels remained unaltered or decreased after MI (▴; n = 21). Values are mean±SEM. *P<0.01 for intra-group comparisons.

Patients with increased s-CTGF levels after PCI displayed significant reduction of indexed LV end-systolic volume (ESVi) during the study period, as compared to patients with unchanged or decreased levels (Fig. 9A). Indexed LV end-diastolic volume (EDVi) was not significantly different between the groups and did not display alterations during follow-up (Fig. 9B). Consistently, LVEF 1 year after primary PCI was significantly improved in patients with increased s-CTGF levels during follow-up (Fig. 9C).

Infarct size decreased significantly during the recovery and study period of both patient sub-groups. Average infarct size, as determined by CMR, on day 2 after PCI was similar between the two groups (15.3±1.5g/m^2^ vs. 16.9±1.8g/m^2^, p = 0.51). After 1 year the infarct size was 8.0±1.1g/m^2^ in patients who responded with increase in s-CTGF levels after MI versus 11.3±1.8g/m^2^ in patients who displayed unaltered or decreased levels during follow-up (Fig. 9D). However, this difference did not reach statistical significance (p = 0.11).

## Discussion

The present study demonstrates increased survival, enhanced infarct healing and attenuated LV remodeling after MI in transgenic mice with cardiac-restricted overexpression of CTGF. In accordance with the animal model, study of patients with ST-elevation MI demonstrated attenuated LV remodeling in the cohort that displayed increasing s-CTGF levels after MI. Thus, the study provides novel translational evidence of enhanced infarct healing and anti-remodeling activities of CTGF.

The area at risk following ligation of the left coronary artery was found to be similar in Tg-CTGF mice and NLC mice. This finding is congruent with previously reported data demonstrating that regional myocardial blood flow was similar in Tg-CTGF mice and NLC mice [7]. Consistent with these findings permanent ligation of the left coronary artery in Tg-CTGF mice and NLC mice generated myocardial infarctions of similar size after 24 hours of ischemia. Thus, the salutary actions of CTGF in post-MI remodeling appear to rely on mechanisms that operate after this time point. First, based on the cardioprotective actions of CTGF due to its stimulation of the Akt/GSK-3β salvage kinase pathway in cardiac myocytes recently reported from our laboratory [7], CTGF might inhibit infarct expansion in the subacute phase after MI. The reduced occurrence of cellular apoptosis in the remote myocardium of Tg-CTGF mice after MI is consistent with this hypothesis. Secondly, the anti-inflammatory properties reported for CTGF may also attenuate pro-inflammatory responses activated by ischemic tissue necrosis and thus, limit infarct expansion [13]. To what extent such anti-inflammatory actions of CTGF is mediated through the Akt/GSK-3 axis is currently unknown. The reduced contents of CD68-positive macrophages, and leucocytes (CD45-positive cells) in general, in the peri-infarct region of Tg-CTGF mice reported in the current study, also support a salutary anti-inflammatory role of CTGF. Consistent with these findings, plasma GDF-15 levels, a TGF-β superfamily cytokine and predictor of developing heart failure [14], as well as plasma CRP levels, were both lower in patients that responded with increasing serum CTGF levels after MI. However, whether the reduced contents of leucocytes 4 weeks after induction of MI, solely reflect anti-inflammatory actions of CTGF or to large extent also enhanced infarct healing and differentiation of scar tissue are yet to be resolved.

Another aspect of CTGF-mediated attenuation of myocardial remodeling is the potential for an effect of CTGF on proliferation of cardiac progenitors or cardiac stem cells, and ultimately, on regeneration of myocardial tissue. Although myocardial tissue contains resident progenitors or stems cells with some regenerative capability, the significance of myocardial regeneration in the natural cause of post-MI healing is limited [15]. However, previous reports have indicated a paracrine factor-stimulated Akt/GSK-3β pathway in proliferation and survival of resident cardiac stem cells [16], [17]. Thus, increased tissue concentrations of paracrine factors that enhance the activity of the Akt/GSK-3β pathway, like that of CTGF, may increase stem cell activity and regeneration of myocardial tissue. In this study we demonstrate increased number of c-kit^+^ cells as well as increased number of cells in mitosis (Ki-67^+^ cells) in the peri-infarct region of Tg-CTGF mice versus that of NLC mice after MI. Of particular interest in this context is a recent report providing evidence of both proliferation of c-kit^+^ stem cells in the peri-infarct region after MI and contribution of these cells to new myocyte formation [18]. A tantalizing interpretation of our data is that the increased contents of c-kit^+^ cells in the peri-infarct region of Tg-CTGF mice may represent increased cardiac stem cell activity. However, c-kit is a cell surface marker not only present on cardiac stem cells. C-kit is widely expressed on hematopoietic stem cells and inflammatory cells and mast cells of hematopoietic origin. On the other hand, the reduced numbers of CD68-positive macrophages and CD45-positive leucocytes (i.e. cells also expressing the surface marker c-kit) in the peri-infarct region of Tg-CTGF would not be consistent with the increased number c-kit^+^ cells in peri-infarct region being inflammatory cells. Thus, the increased number of c-kit^+^ cells in the peri-infarct region of Tg-CTGF mice may represent bona fide cardiac stem cell activity. Supporting a putative role of CTGF-mediated proliferation of cardiac stem cells/cardiac progenitors after MI are recent data demonstrating that CTGF stimulates proliferation of cardiosphere-derived cells *in vitro* [19]. However, the implications of the latter findings, i.e. to what extent CTGF may stimulate regeneration of myocardial tissue *in vivo* still remain to be settled.

This study also indicates that reduced cellular apoptosis of the remote myocardium Tg-CTGF mice may contribute to reduced loss of myocardial tissue reflecting in decreased LV dilatation and cardiac myocyte hypertrophy. Indeed, Tg-CTGF mice disclosed reduced cross-sectional area of cardiac myocytes four weeks after MI. However, the latter finding could also be due to direct CTGF-engendered inhibition of myocardial hypertrophy through induction of anti-hypertrophic gene programs previously reported in Tg-CTGF mice [7]. Thus, inhibition of myocardial hypertrophy *per se* after MI may also contribute to attenuated LV remodeling. The slight increase of microvessel density in the peri-infarct region of Tg-CTGF mice 4 weeks after MI compared with that of NLC mice is consistent with a previous report from our laboratory, demonstrating increased microvessel densities in myocardial tissue of Tg-CTGF mice not subjected to myocardial infarction [7]. The significance of such an increase in myocardial microvessel densities of Tg-CTGF mice is uncertain. However, despite the fact that myocardial blood flow of Tg-CTGF mice was not found to be statistically different from that of NLC mice [7], the increase of myocardial microvessel densities of Tg-CTGF mice may provide shorter distances of oxygen diffusion in the tissue, and thus, improved transport of oxygen to the cells. The latter would be particularly relevant under the increased workload of the remaining myocardial tissue after myocardial infarction and would also be consistent with the reduced incidence of cellular apoptosis in remote myocardial tissue of Tg-CTGF versus that of NLC mice reported in this study.

Mice with cardiac-restricted overexpression of CTGF displayed subtle increase of myocardial collagen contents compared with non-transgenic control mice [7]. Furthermore, the CTGF-engendered increase of myocardial collagen contents was minor compared with that of non-ischemic myocardial tissue in ischemic heart failure. Indeed, the increase of myocardial collagen contents in ischemic heart failure was less in Tg-CTGF mice that that in non-transgenic control mice, consistent with the attenuated signals for LV remodeling under cardiac exposure to CTGF. An obvious interpretation of the data is that CTGF, even under the settings of ischemic heart failure, is not a major driver of myocardial fibrosis. Such an interpretation may seem at odds with reports demonstrating that myocardial CTGF expression is associated LV remodeling in heart failure [20], [21], [22]. However, although myocardial CTGF expression may reflect pathologic LV remodeling and myocardial fibrosis, no studies have yet reported that CTGF is a principal driver of fibrosis. Thus, a function of CTGF as a marker of myocardial damage and remodeling in heart failure is not in contrast to a cardioprotective function of CTGF.

Increase of s-CTGF levels in patients after MI was associated with similar anti-remodeling effects as in Tg-CTGF mice. Despite the fact that relatively few patients were included, this study represents a homogenous cohort of patients with acute ST-elevation MI, due to single vessel thrombus, successfully revascularized by PCI. The use of repeated CMR examinations at discrete time-points allowed a precise description of both infarct healing and LV function. Although the s-CTGF levels were not statistically different at any time point during follow-up among the groups, the statistical analysis revealed significant intra-patient variation during infarct healing with the patient cohort segregating into two sub-groups; one in which s-CTGF levels increased after MI, and the other in which s-CTGF levels remained unaltered or decreased. These striking differences in the intra-patient responses of s-CTGF levels after MI allowed analyses of the putative relations of quantitative parameters of LV remodeling and function to that of the course of s-CTGF levels after MI. Interestingly, only patients who responded with increase in s-CTGF after MI displayed attenuated LV remodeling and improved recovery of LV function. The wide inter-patient variations in circulating CTGF levels are consistent with previous reports and could have multiple explanations [10], [23]. On the other hand, the distinct intra-patient responses of s-CTGF levels after MI could be due to single nucleotide polymorphisms in the CTGF gene. Indeed, previous reports have disclosed single nucleotide polymorphisms in the promoter region of CTGF at binding sites for transcription factors [24], [25]. Although s-CTGF levels were not studied in relation to these specific polymorphisms, patients homozygous for the G allele at -945 exhibited loss of Sp1 binding to the promoter and lack of Sp1-mediated repression of the promoter [24]. Thus, the striking differences in s-CTGF responses to MI and post-MI remodeling in patients may be due to polymorphisms in the promoter of CTGF.

In conclusion, the congruent findings of attenuated LV remodeling after MI in mice with cardiac-restricted overexpression of CTGF as well as in patients that respond with increased s-CTGF levels after MI, support a beneficial role of CTGF in LV remodeling and functional recovery of the heart after MI mediated by attenuation of inflammatory responses and inhibition of apoptosis.
